# Coping and risk perception during the COVID-19 pandemic in type 2 diabetes: Does it influence metabolic control?

**DOI:** 10.1371/journal.pgph.0002793

**Published:** 2024-02-13

**Authors:** María Teresa Alcántara-Garcés, Alejandra Monserrat Rodríguez-Ramírez, Ana Cristina García-Ulloa, Mario García-Alanis, Gabriela Nazareth Martínez-Reyes, Lupita Paola Del Moral Vidal, Rodrigo Eduardo Arizmendi-Rodríguez, Sergio Hernández-Jiménez, Paloma Almeda-Valdes

**Affiliations:** 1 Centro de Atención Integral del Paciente con Diabetes (CAIPaDi) Instituto Nacional de Ciencias Médicas y Nutrición Salvador Zubirán, Mexico City, Mexico; 2 Neurology and Psychiatry Department, Instituto Nacional de Ciencias Médicas y Nutrición Salvador Zubirán, Mexico City, Mexico; 3 Endocrinology and Metabolism Department, Instituto Nacional de Ciencias Médicas y Nutrición Salvador Zubirán, Mexico City, Mexico; 4 Metabolic Diseases Research Unit, Instituto Nacional de Ciencias Médicas y Nutrición Salvador Zubirán, Mexico City, Mexico; T D Medical College, INDIA

## Abstract

Diabetes and poor glycemic control are significant predictors of severity and death in the COVID-19 disease. The perception of this risk in individuals with type 2 diabetes (T2D) could modify coping styles, leading to behaviors associated with better self-care and metabolic control. Theoretically, active coping is associated with better glycemic control in patients with T2D. Nonetheless, information during extreme risk like the COVID-19 pandemic is still limited. Our objective was to evaluate the association between coping styles and risk perception in the COVID-19 pandemic and the change in metabolic parameters. This is a prospective study that included individuals with T2D treated in a tertiary care center during the COVID-19 outbreak who returned to follow-up one year later. We assessed coping styles and risk perception with the Extreme Risk Coping Scale and the risk perception questionnaire. Clinical characteristics and metabolic parameters were registered in both visits. Groups were compared using Kruskal Wallis tests, and changes in metabolic parameters were assessed with Wilcoxon rank sum tests. Our sample included 177 participants at baseline, and 118 concluded the study. Passive coping was more frequent in women. Low-risk perception was associated with higher age, lower psychiatric comorbidities, and lower frequency of psychiatric treatment compared with other risk perception groups. Patients with active coping plus high-risk perception did not have a change in metabolic parameters at follow-up, whereas patients with other coping styles and lower risk perception had an increase in total cholesterol, LDL-cholesterol, and triglycerides. There were no differences by coping group or by risk perception in glycemic control.

## Introduction

Patients with chronic medical conditions such as type 2 diabetes (T2D) have been identified as a population with worse prognosis when infected with SARS-CoV-2 [1]. Previous data reported higher odds of mortality and complications in individuals with T2D and higher glycemia during hospitalization for COVID-19 [2]. This information was massively disseminated by media and governments around the world to alert the populations at risk and promote the prevention of infection and selfcare. Nonetheless, the impact of such information in each person relays at least in two factors: coping styles and risk perception. These personal traits may lead to a wide spectrum of behaviors in response to adversity going from an extreme concern for health, to a complete denial [3, 4]. Hence, there is a need for evidence on how these psychobiological factors influence metabolic control of T2D during the COVID-19 pandemic.

As a result of the COVID-19 pandemic, a constant source of stress was perceived, and daily life changed with a psychological impact and necessity to adapt [5]. Coping styles are sets of skills defined as cognitive and behavioral efforts to relieve emotional stress and modify the source of threat, achieve adaptation, and preserve functionality in new circumstances [6, 7]. Lazarus and Folkman classified coping in emotion-focused coping (efforts directed at regulating negative emotional states) and in problem-focused coping (efforts directed at the source of stress to modify or eliminate it) [6]. Moss and Billings describe an active coping (logical analysis, positive feedback, seeking support, problem solving) *vs* passive or avoidance coping (cognitive avoidance, acceptance/resignation, search for alternative gratifications, negative emotional discharge) [7]. In T2D, an active coping style is associated with better glycemic control [8, 9]. The impact of coping styles in metabolic control in T2D during the COVID-19 pandemic has yet to be dilucidated.

Risk perception is a subjective concern about vulnerability through interpretation of the world. It is based on intrapersonal and interpersonal factors and has a positive association with the feeling of stress [10–12]. Risk perception has been studied in previous pandemics and catastrophic risk situations. In general, population with a high-risk perception have a negative affective response [13], higher stress and passive coping [14]. In individuals with T2D, there is no previous research evaluating risk perception in situations that go beyond the classic hazard, called extreme risk, such as the COVID-19 pandemic [15]. Coping styles and risk perception might impact the selfcare of T2D patients with consequences in metabolic control. This study aims to evaluate the association between coping styles and risk perception during the COVID-19 pandemic with changes in metabolic parameters in individuals with T2D.

## Materials and methods

We conducted a prospective study of T2D individuals treated in a tertiary care center during the COVID-19 outbreak (January-June 2020) follow-up them one year later (January-June 2021) in Mexico City.

### Study population

We included patients over 18 years old, with previously confirmed T2D diagnosis. We excluded patients that were not able to answer the instruments, pregnant women, and with other diabetes types. The study was approved by the Institutional Ethics and Research Committees from the Instituto Nacional de Ciencias Médicas y Nutrición Salvador Zubirán in Mexico City (Ref. 3399). All participants who agreed to participate signed an informed consent form. This research was conducted according to the Declaration of Helsinki Ethical principles.

### Procedures

Diagnosis of T2D was established according to the American Diabetes Association (ADA) criteria (HbA1c ≥ 6.5% or fasting plasma glucose ≥ 126 mg/dL or random plasma glucose ≥ 200 mg/dL with symptoms) [16]. Clinical characteristics (sex, age, time since T2D diagnosis, medical comorbidity, psychiatric comorbidity, oral antidiabetics, insulin therapy, psychiatric treatment, exercise, diet, and adherence), metabolic and anthropometric parameters (HbA1c, lipid profile, body mass index) were registered at baseline by an endocrinologist and a nutritionist. In the baseline evaluation, a liaison psychiatrist registered the psychiatric comorbidities, psychopharmacological treatment at enrollment, and obtained measures of coping styles and risk perception.

Coping styles were assessed using the Extreme Risk Coping Scale, in its Mexican version (alpha-Cronbach 0.81) [17]. The answers are in a Likert scale to identify active coping (behaviors to direct action on resolving the problem, search of information to resolve a problem, strategies to anticipate a disaster and control of oneself and circumstances), passive coping (behaviors of rejection and denial of the problem, withdrawal or avoiding the problem, and passive acceptance of the problem), mixed coping (active and passive coping), and indefinite coping (neither active nor passive coping). Risk perception (RP) was assessed by two questions: a personal rating of the probability of being affected by COVID-19, and a personal rating of probability of being infected by COVID-19 [18]. The answers identify three groups: low RP, middle RP and high RP.

Blood samples were taken for measurement of lipid profile (using colorimetric methods, SYNCHRON CX System), HbA1c (using HPLC method, Bio-Rad Variant II Turbo HbA1c Kit 2) and body composition was assessed by bioimpedance (body composition analyzer JAWON medical ioi353) at baseline and follow-up.

### Statistical analysis

Normal distribution was assessed with the Kolmogorov-Smirnoff test. We present the data with means and standard deviation or median with interquartile range (IQR 25–75) accordingly. Categorical variables are reported in frequencies and percentages. A comparative analysis of clinical characteristics and metabolic parameters at baseline and follow-up was performed by coping styles and risk perception. Continuous variables were compared with Kruskal Wallis tests and Student’s T-test or Mann-Whitney U test according to the distribution and number of groups. We used Wilcoxon rank sum test to compare changes in HbA1c, total cholesterol, LDL-cholesterol, HDL-cholesterol, non-HDL cholesterol, triglycerides, and BMI. The comparison between categorical variables was assessed using the Chi-square test. We used STATA 14.0 (StataCorp. 2014. Stata Statistical Software: Release 14. College Station, TX: StataCorp LP).

## Results

**Fig 1:**
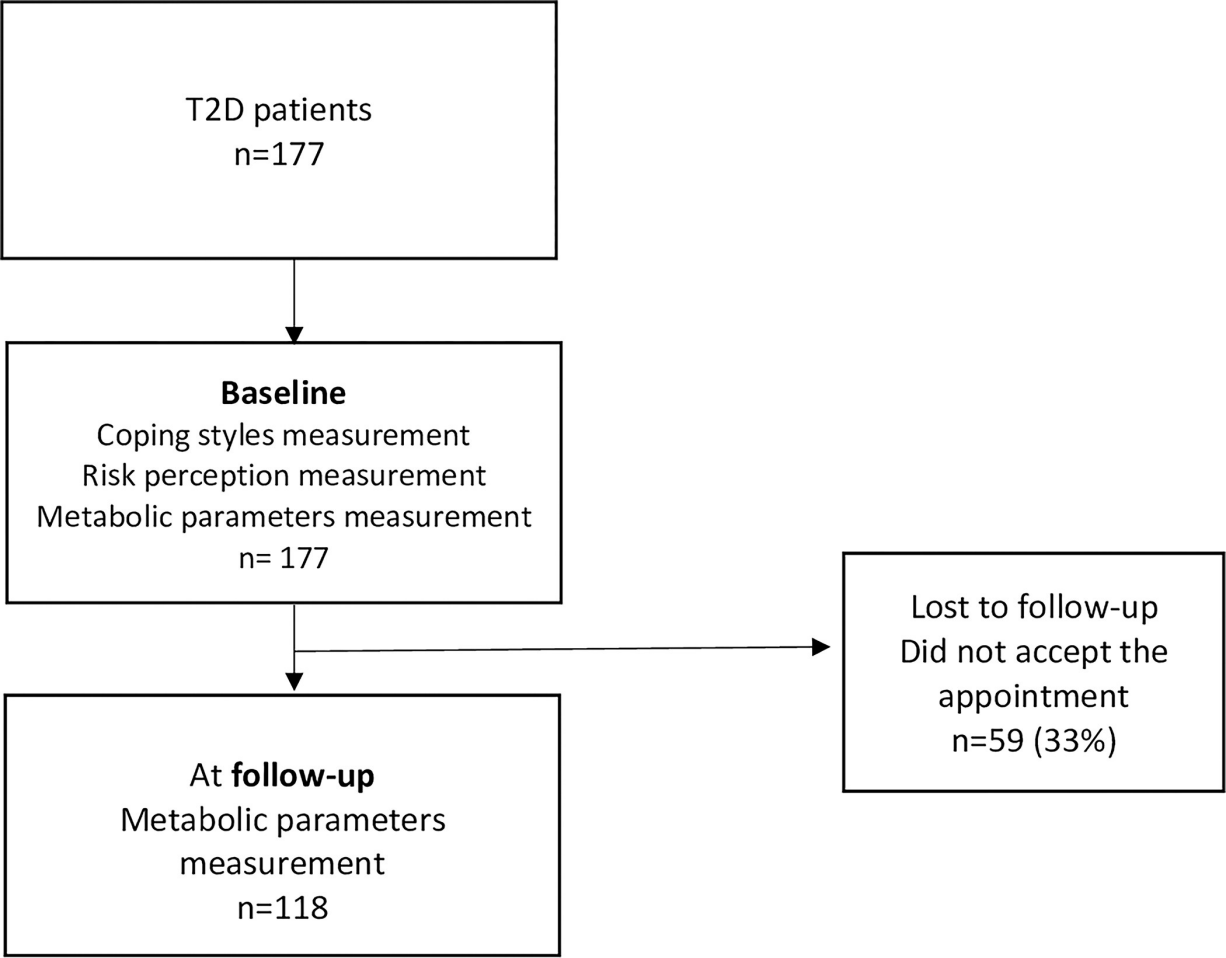
Flowchart of participants.

At baseline, our sample included 177 participants (Fig 1), 65.5% were women, with a median age of 56 (49–62) years. More than half of the participants had less than 5 years since T2D diagnosis. We identified 92% of subjects with a medical comorbidity and 43.5% had a psychiatric comorbidity (17.7% with affective disorders, 17.5% with anxiety disorders, 15.4% personality disorders, 10.7% with sleep disorders, 8.5% substance use disorders, and 6.3% with eating disorders). The percentage of participants that had a follow-up visit was 66.7% (n = 118), 65.3% were women, with a median age 53 (48–61) years, 91.5% had any medical comorbidity, with a higher percentage having psychiatric comorbidity (53.4%) compared with the participants at baseline (*p* = 0.001).

In the total population total cholesterol increased from 165 mg/dL (145–198) to 177 mg/dL (153–215), *p* = 0.007), LDL-cholesterol from 95 mg/dL (78–119) to 106 mg/dL (83–131), *p* = 0.01), and triglycerides from144 mg/dL (107–202) to 168 mg/dL (113–239), *p* = 0.01) at follow up. There was no change in HbA1c (6.9% (6.1–8.6) *vs* 7.1% (6.3–8.8), *p* = 0.224), HDL-cholesterol (44 mg/dL (38–53) *vs* 42 mg/dL (37–50), *p* = 0.597), non-HDL cholesterol (123 mg/dL (99–157) *vs* 131 mg/dL (108–170), *p* = 0.104) or BMI (28.9 kg/m^2^ (26–32.6) *vs* 28.8 kg/m^2^ (25.9–31.9), *p* = 0.324). Pharmacological antidiabetic treatment (oral antidiabetics 92.6% *vs* 92.4%, *p* = 0.860, insulin therapy 24.3% *vs* 22.9%, *p* = 0.687); psychiatric treatment 23.1 *vs* 22.9%, *p* = 0.758; exercise performance 53.1% *vs* 53.4%, *p* = 0.915; diet adherence 44.6 *vs* 44.9%, *p* = 0.915; treatment adherence 79.1% *vs* 79.6, *p* = 0.555 did not change.

### Clinical and metabolic characteristics by coping styles

We divided our sample by coping style in four groups: active, passive, mixed, and undefined coping (Table 1). The most frequent style in our population was active coping, representing 30% of the baseline sample and 35% of patients who completed the follow-up. Seventy seven percent of patients with active coping completed the study, whereas individuals with other coping styles had lower permanence (passive 60%, mixed 71%, undefined 59%).

**Table 1:** Comparative analysis of clinical characteristics according to coping styles at baseline and follow-up.

	Baseline n = 177					Follow-up n = 118				
Variable	Active coping	Passive coping	Mixed coping	Indefinite coping	*p* value	Active coping	Passive coping	Mixed coping	Indefinite coping	*p* value
	n = 54	n = 48	n = 21	n = 54		n = 42	n = 29	n = 15	n = 32	
Sex n (%)					**0.028**					
Female	30 (55.6)	35 (72.9)	10 (47.6)	41 (75.9)		21 (50)	21 (72.4)	6 (40)	24 (75)	**0.027**
Male	24 (44.4)	13 (27.1)	11 (52.4)	13 (24.1)		21 (50)	8 (27.6)	9 (60)	8 (25)	
Age (years) Median (IQR)	57 (51–62)	55 (47–65)	56 (48–61)	56 (48–61)	0.769	53 (50–61)	52 (47–61)	55 (48–61)	53 (47–60)	0.897
Time since T2D diagnosis (%)										
< 5 years	28 (51.8)	30 (62.5)	11 (52.4)	32 (59.2)	0.213	25 (59.5)	20 (67)	8 (53.3)	23 (71.9)	0.146
5–9 years	16 (29.6)	6 (12.5)	4 (19.0)	14 (26.0)		11 (26.2)	2 (7)	4 (26.7)	7 (21.9)	
10–14 years	4 (7.4)	2 (4.1)	4 (19.0)	4 (7.4)		3 (7.1)	2 (7)	3 (20)	1 (3.1)	
≥ 15 years	6 (11.1)	10 (20.8)	2 (9.5)	4 (7.4)		3 (7.1)	5 (17.2)	0 (0)	1 (3.1)	
Medical comorbidity n (%)	49 (90.7)	42 (87.5)	19 (90.5)	53 (98.1)	0.231	37 (88.1)	26 (89.7)	14 (93.3)	31 (96.9)	0.567
Psychiatric comorbidity n (%)	22 (40.8)	25 (52.1)	9 (42.9)	21 (38.9)	0.559	17 (40.5)	19 (65.5)	9 (60)	18 (56.2)	0.187
Oral antidiabetics n (%)	52 (96.3)	44 (91.7)	20 (95.2)	48 (88.9)	0.751	40 (95.2)	27 (93.1)	14 (93.3)	28 (87.5)	0.881
Insulin therapy n (%)	8 (14.8)	15 (31.2)	5 (23.8)	15 (27.8)	0.516	5 (11.9)	7 (24.1)	3 (20)	12 (37.5)	0.214
Psychiatric treatment n (%)	9 (16.7)	16 (33.3)	2 (9.5)	14 (25.9)	0.137	6 (14.9)	9 (31)	2 (13.3)	10 (31.2)	0.226
Exercise adherence n (%)	34 (63.0)	21 (43.8)	10 (47.6)	29 (53.7)	0.256	26 (61.9)	14 (48.3)	7 (46.7)	16 (50)	0.576
Diet adherence n (%)	34 (63.0)	16 (33.3)	10 (47.6)	19 (35.2)	**0.008**	25 (59.2)	12 (41.4)	7 (46.6)	9 (28.1)	0.060
Medication adherence n (%)	46 (85.1)	36 (75.0)	16 (76.2)	42 (77.8)	0.602	37 (88.1)	22 (75.9)	10 (66.7)	25 (78.1)	0.294
HbA1c (%) Median (IQR)	6.5 (6.1–7.8)	6.9 (6–8.5)	7.2 (6–10.2)	6.9 (6.1–8.6)	0.246	6.3 (6.9–8.2)	6.8 (6.1–8.1)	8.4 (6–10.1)	7.5 (6.7–8.8)	0.230
Total-cholesterol (mg/dL) Median (IQR)	162 (142–194)	167 (149–201)	177 (139–199)	165 (140–201)	0.661	175 (155–206)	182 (161–228)	177 (157–243)	171 (140–221)	0.380
LDL-cholesterol (mg/dL) Median (IQR)	93 (78–119)	95 (80–114)	102 (79–112)	96 (75–122)	0.856	108 (87–132)	111 (93–136)	97 (82–114)	91 (80–129)	0.558
HDL-cholesterol (mg/dL) Median (IQR)	45 (40–54)	45 (36–53)	42 (36–50)	43 (37–51)	0.464	46 (40–50)	43 (37–59)	42 (34–50)	39 (36–43)	**0.022**
Non-HDL-cholesterol (mg/dL)	120 (98–156)	123 (104–157)	128 (100–156)	119 (95–158)	0.724	127 (112–157)	134 (107–163)	134 (118–198)	132 (106–179)	0.626
Triglycerides (mg/dL) Median (IQR)	146 (104–173)	135 (101–209)	140 (119–185)	154 (109–215)	0.679	161 (113–227)	153 (99–248)	195 (136–347)	172 (113–232)	0.401
Body mass index (kg/m^2^) Median (IQR)	29.2 (27.2–33.6)	28.9 (25.6–32.5)	28.5 (26.2–31.4)	28.1 (25.4–32.7)	0.819	29.9 (26–32.8)	29.4 (27.6–31.4)	28.2 (27.3–31.9)	27.8 (24.8–34.8)	0.772

Abbreviations: IQR, interquartile range; T2D, Type 2 Diabetes; HbA1c, glycated hemoglobin; LDL, Low Density Lipoprotein; HDL, High Density Lipoprotein; Non-HDL, Non-High-Density Lipoprotein.

We found a higher percentage of women in the passive coping group 72.9% *vs* 27.1, *p*<0.05). The active coping group reported a higher percentage of diet adherence (63% *vs* 33.3% in passive coping, 47.6% in mixed coping, and 53.7% in undefined coping; *p* = 0.008). In individuals who returned to follow-up metabolic and anthropometric characteristics did not change (Table 1).

### Clinical and metabolic characteristics by risk perception

We identified three groups according to their RP: low RP (n = 64, 36.1%), middle RP (n = 58, 32.7%) and high RP (n = 55, 31%). The low RP group were older (60 years (52–64), 52 (47–60) and 56 (48–62); *p* = 0.006), had a lower percentage of psychiatric comorbidity (30.2%, 46.5% and 54.5%; *p* = 0.02), and lower frequency of psychiatric treatment (7.9%, 31% and 32.7%; *p* = 0.001) *vs* the other RP groups.

In the sample with a follow-up in the low RP group the HDL cholesterol reduced (-2 mg/dL (-6 a 1), -0.5 mg/dL (-3 a 4) in the middle RP, and 3 mg/dL (-4 a 7) high RP; *p* = 0.02) and no changes were identified over other metabolic parameters as shown in Table 2.

**Table 2:** Comparative analysis of clinical characteristics between risk perception at baseline and follow-up.

	Baseline				Follow-up			
	n = 177				n = 118			
Variable	Low RP	Middle RP	High RP	*p* value	Low RP	Middle RP	High RP	*p* value
	n = 64	n = 58	n = 55		n = 39	n = 42	n = 37	
Sex n (%)								
Female	39 (60.3)	38 (65.5)	39 (70.9)	0.483	19 (47.4)	25 (59.5)	28 (75.7)	**0.042**
Male	25 (39.7)	20 (34.5)	16 (29.1)		20 (52.6)	17 (40.5)	9 (24.3)	
Age (years) Median (IQR)	60 (52–64)	52 (47–60)	56 (48–62)	**0.006**	58.5 (52–62)	51(45–56)	53 (48–60)	**0.004**
T2D diagnosis (%)								
< 5 years	36 (55.5)	37 (63.8)	29 (52.7)	0.507	26 (68.4)	27 (64.3)	23 (62.2)	0.148
5–9 years	14 (22.2)	10 (17.2)	16 (29.1)		9 (23.7)	6 (14.3)	9 (24.3)	
10–14 years	7 (11.1)	2 (3.5)	4 (7.3)		2 (5.3)	2 (4.8)	4 (10.8)	
≥ 15 years	7 (11.1)	9 (15.5)	6 (11)		1 (2.6)	7 (16.7)	1 (2.7)	
Medical comorbidity n (%)	58 (92.1)	51 (87.9)	53 (96.4)	0.250	35 (92.1)	36 (85.7)	36 (97.3)	0.182
Psychiatric comorbidity n (%)	19 (30.2)	27 (46.5)	30 (54.5)	**0.022**	16 (42.1)	23 (54.8)	23 (62.2)	0.211
Oral antidiabetics n (%)	58 (92.1)	53 (91.4)	52 (94.6)	0.441	35 (92.1)	39 (92.9)	34 (91.9)	0.770
Insulin therapy n (%)	11 (17.5)	16 (27.6)	16 (29.1)	0.392	4 (10.5)	14 (33.3)	9 (24.3)	**0.046**
Psychiatric treatment n (%)	5 (7.9)	18 (31)	18 (32.7)	**0.001**	3 (7.9)	14 (33.3)	10 (27)	**0.022**
Exercise n (%)	38 (60.3)	32 (55.2)	24 (43.6)	0.184	24 (63.1)	23 (54.8)	16 (43.2)	0.222
Diet n (%)	32 (50.8)	24 (41.4)	22 (40)	0.427	21 (55.3)	17 (40.5)	14 (37.8)	0.256
Adherence n (%)	52 (82.5)	46 (79.3)	42 (76.4)	0.708	30 (78.9)	37 (88.1)	27 (73)	0.233
HbA1c (%) Median (IQR)	6.9 (5.9–8.6)	6.9 (6.3–9.1)	6.7 (6.1–8.4)	0.430	7.2 (6.4–9)	7.4 (6.6–9.1)	6.9 (6–8.1)	0.246
Total-cholesterol (mg/dL) Median (IQR)	166 (139–201)	158 (141–187)	175 (145–214)	0.102	176 (157–208)	168 (153–210)	190 (151–228)	0.616
LDL-cholesterol (mg/dL) Median (IQR)	94 (75–124)	91 (80–104)	102 (80–132)	0.285	102 (85–121)	103 (86–123)	123 (77–145)	0.634
HDL-cholesterol (mg/dL) Median (IQR)	45 (38–53)	44 (37–54)	43 (37–51)	0.666	40 (36–50)	43 (38–52)	43 (38–52)	0.399
Non-HDL-cholesterol (mg/dL) Median (IQR)	126 (94–157)	112 (99–135)	129 (99–168)	0.143	127 (110–159)	123 (108–156)	140 (101–190)	0.473
Triglycerides (mg/dL) Median (IQR)	145 (114–209)	131 (102–171)	143 (101–223)	0.455	195 (136–260)	159 (112–246)	140 (99–200)	0.105
Body mass index (kg/m^2^) Median (IQR)	28.9 (26.4–32.7)	28.2 (25.5–31.3)	30 (26.4–33.4)	0.370	28.9 (25.7–33.6)	27.9 (25.5–30.4)	30.5 (27–34.8)	0.331

Abbreviations: IQR, interquartile range; RP, Risk Perception; T2D, Type 2 Diabetes; HbA1c, glycated hemoglobin; LDL, Low Density Lipoprotein; HDL, High Density Lipoprotein; Non-HDL, Non-High-Density Lipoprotein.

### Comparison of clinical and metabolic characteristics by coping styles and risk perception

**Fig 2:**
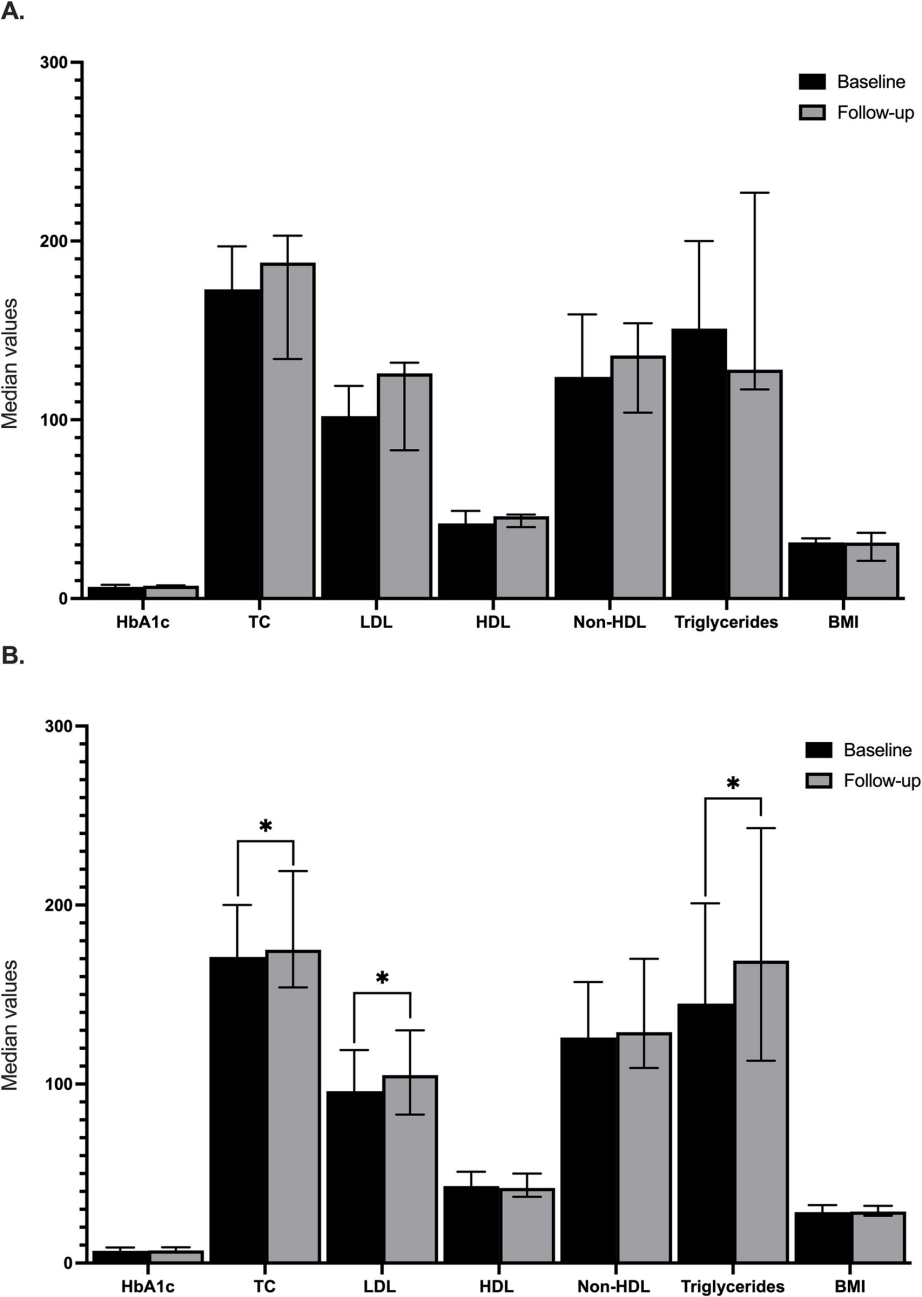
Comparative analysis between baseline and follow-up metabolic parameters. A. Patients with active coping plus high-risk perception. B. Patients with other coping styles and perception risk.

We compared the subgroup of patients with an active coping *plus* high-RP (n = 13 at baseline and n = 9 at follow-up) *versus* the rest of coping styles and RP (n = 164/n = 109). This group had a higher percentage of adherence to diet (76.9% *vs* 42.15, *p* = 0.01). We identified that in patients with an active coping *plus* high RP metabolic parameters remained stable (Fig 2A), whereas in the rest of patients with different coping styles and lower RP total cholesterol (171 mg/dL (145–200) *vs* 175 mg/dl (154–219); *p* = 0.014), LDL-cholesterol (96 mg/dl (78–119) *vs* 105 mg/dl (83–130); *p* = 0.021) and triglycerides (145 mg/dl (105–201) *vs* 169 mg/dl (113–243); *p* = 0.010) increased (Fig 2B).

## Discussion

This longitudinal study explored the impact of coping styles and RP during the COVID-19 outbreak in metabolic parameters of patients with T2D. We identified differences in sex, age, diet adherence, psychiatric comorbidity, treatment and metabolic parameters among coping styles and risk perception groups. Prior to COVID-19 pandemic, studies reported the association between good glycemic control and active coping [8, 9, 19]. A cross-sectional observational study included patients with type 1, type 2, and gestational diabetes in Saudi Arabia. The authors reported self-care behaviors as an adaptative coping and 78.8% measured their blood glucose and only 36% of patients reported adequate blood glucose levels (81–120 mg/dL) [20]. However, these studies were carried out without an extreme global threat that could modify coping. Our interest was to explore coping styles and COVID-19 risk perception.

Previous studies have reported coping styles differences by sex in patients with T2D. In agreement with this information, our study identified that women had more passive coping whereas men had higher frequency of active coping. A Swedish study reported resignation, protests, and isolation as part of passive coping in women [21]. In a similar manner, our female population reported passive coping strategies like rejection, denial, and withdrawal. Sociocultural and gender stereotypes may explain the differences [22, 23]. We identified an association between active coping and higher diet adherence, an important self-care behavior to achieve the glycemic control goal. Even so, ours results about metabolic parameters were similar between coping styles and no differences were identified in HbA1c. These findings are different from those reported before COVID-19 in other studies. An Iranian cross-sectional study reported an association with lower HbA1c and higher self-care, positive coping styles and greater social support [19]. We hypothesized that the differences in our results are related with sociocultural differences, a higher percentage of patient with less than 10 years since T2D diagnosis with a better pancreatic reserve, and the losses at follow-up that might limit the power of this study.

Differences according to COVID-19 RP were identified, low RP was associated with older age, male sex, lower psychiatric comorbidity, lower psychiatric treatment, and lower insulin therapy. Our results coincide with a German study that evaluated the RP during the pandemic in which older patients and men estimated a lower perception of risk during the COVID-19 pandemic [24]. A possible explanation for this association is the previous experience during the H1N1 pandemic in 2009 that older patients may had. Also, older patients could had taken greater actions to avoid SARS-CoV-2 infection, were more protected by the government and their family than younger patients, and received prioritized immunity by vaccine application, all this may have influenced their RP. Risk perception is a complex psychological construct with the potential to modify self-care in patients with T2D, but the results of RP can be strongly different between patients. Theoretically, RP function as a trigger for precautionary action [15]; nonetheless, our results showed similar percentages of diet adherence, exercise, medical comorbidities, and metabolic parameters between groups. We considered that these results can be explained by the losses at follow-up that reduced the sample size. Interestingly, differences in mental health were identified, lower psychiatric comorbidity and lower psychiatric treatment were associated with low RP and high RP with higher psychiatric treatment. Previous studies identified preliminary significantly associations between higher RP of COVID-19 with less positive emotions (relaxed, calm, content, happy, excited) or more negative emotions (anxious, nervous, depressed, exhausted, lonely, bored) [25]. Thus, mental health attention is still fundamental after COVID-19 pandemic.

Finally, we identified a subsample of patients with active coping plus high-risk perception that had stable metabolic parameters after COVID-19 outbreak compared with patients with other coping styles and lower RP. This difference can be an important consideration about the interrelation of psychologic characteristics that could be related in behaviors influencing self-care and the impact over chronic illness control.

We acknowledge some limitations in our study, such as the losses at follow-up, the higher percentage of female subjects in our study and a high percentage of patients with less than 10 years since T2D diagnosis. Nonetheless, this study provides information about the influence of psychologic phenomena on metabolic control during a health crisis that changed our world. The strengths of this study are the exploration of two different personal characteristics in independent and combined manner, and a longitudinal design that give us a wider vision of our population.

## Conclusion

This study explored coping styles and risk perception during COVID-19 outbreak in patients with type 2 diabetes and compared clinical characteristics and metabolic parameters. Our findings reported higher adherence of diet in patients with active coping, higher psychiatric comorbidity in patients with high-risk perception and stable metabolic parameters in patients with active coping plus high-risk perception. Coping styles and risk perception were associated with different clinical characteristics and metabolic parameters, which highlight the importance of mental health care in patients with type 2 diabetes.
